# Transient changes during microwave ablation simulation : a comparative shape analysis

**DOI:** 10.1007/s10237-022-01646-6

**Published:** 2022-10-26

**Authors:** Dale Kernot, Jimmy Yang, Nicholas Williams, Tudor Thomas, Paul Ledger, Hari Arora, Raoul van Loon

**Affiliations:** 1 School of Engineering and Applied Sciences, Faculty of Science and Engineering, Swansea University, Fabian Way, Swansea, Glamorgan SA1 8EN UK; 2 Olympus Surgical Technologies Europe, Fortran road, Cardiff, Glamorgan CF3 0LT UK; 3 School of Computing and Mathematics, Keele University, Keele, Staffordshire ST5 5BG UK

**Keywords:** Microwave ablation (MWA), Bioheat, Hyperthermal treatment, Numerical simulation, Shape analysis, Temperature sensitivity

## Abstract

Microwave ablation therapy is a hyperthermic treatment for killing cancerous tumours whereby microwave energy is dispersed into a target tissue region. Modelling can provide a prediction for the outcome of ablation, this paper explores changes in size and shape of temperature and Specific absorption rate fields throughout the course of simulated treatment with different probe concepts. Here, an axisymmetric geometry of a probe embedded within a tissue material is created, solving coupled electromagnetic and bioheat equations using the finite element method, utilizing hp discretisation with the NGSolve library. Results show dynamic changes across all metrics, with different responses from different probe concepts. The sleeve probe yielded the most circular specific absorption rate pattern with circularity of 0.81 initially but suffered the largest reduction throughout ablation. Similarly, reflection coefficients differ drastically from their initial values, with the sleeve probe again experiencing the largest change, suggesting that it is the most sensitive the changes in the tissue dielectric properties in these select probe designs. These collective characteristic observations highlight the need to consider dielectric property changes and probe specific responses during the design cycle.

## Introduction

Currently, in medicine, a common method of treatment after detection of a focal tumour is surgical removal of diseased tissue. However, open surgery can bring adverse affect that has driven the search alternatives. Thermal ablation therapy is a procedure that aims to destroy an area of tissue by application of cytotoxic temperatures. The temperature change is targeted at an area within and surrounding the tumour itself, with the objective of removing the tumour in addition to a portion of surrounding healthy tissue which can be compared to the resection margin Brace (2011a).

One thermal ablation treatment is MWA, which is based on the fundamental principle of a microwave frequency energy source dispersing into a local tissue region, heat is generated through the dipole rotation of molecules in the presence of this high frequency oscillating electromagnetic field. Microwaves are able to propagate into the target tissue through the use of an interstitial antenna, acting to provide the link between the microwave source and tissue material. The MWA method can rapidly heat a large volume of tissue, to higher temperatures than more established thermal therapies Poulou et al. (2015).

Predicting the progression of ablation during one of these procedures is of high value in designing equipment and planning patient-specific care. Numerical modelling provides a highly controlled environment to simulate and evaluate device performance quickly. However, accurate prediction of the temperature distribution around the probe throughout ablation is challenging due to numerous changes occurring as the tissue heats up Rossmanna and Haemmerich (2014). Mainly this requires the highly temperature-sensitive nature of biological materials to be accounted for, the influence of changes in tissue dielectric and thermal properties on the development of ablation can be investigated by creation of a simplified model that takes advantage of the symmetry of probe designs, but makes the assumption that the tissue, in which it is embedded, is also axis-symmetric with no large vessels within the domain Prakash (2010); Bertram et al. (2006).

**Fig. 1:**
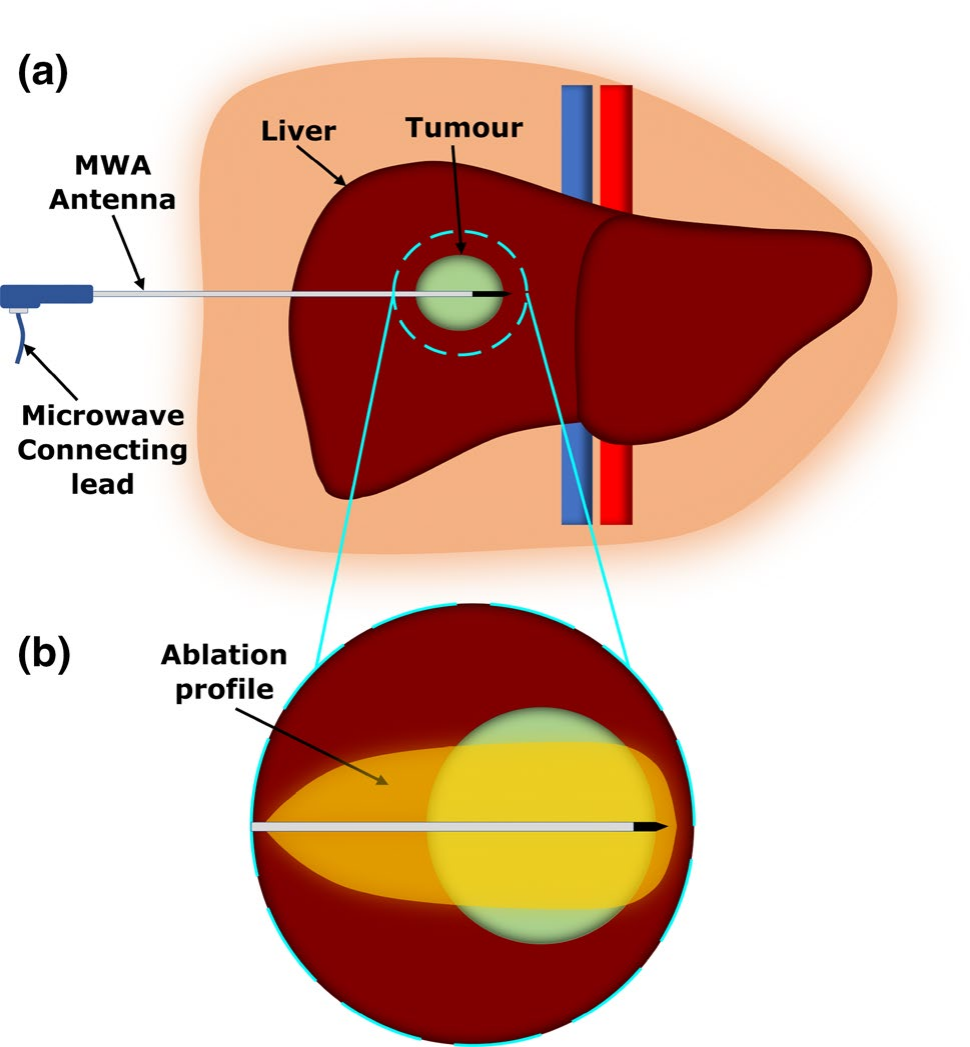
**a** The MWA probe is inserted into the tumour embedded within the liver, treatment can then begin and a ablated area is created from the increased temperature. **b** The shape of this ablation may vary from the target tumour, unceasing the likelihood of collateral damage to neighbouring healthy tissue, or leaving viable tumour behind

One of the disadvantages of MWA is the characteristically elliptical or tear-drop shape of the electric field (therefore, also Specific Absorption Rate (SAR)) and subsequently the temperature profile, which presents a problem if the tumour is spherical in shape. An elliptical or tear-drop-shaped ablation area leaves potential for incomplete heating of the target tumour, or excessive damage to otherwise healthy tissues away from the targeted area illustrated in Fig. 1.The shape and extent of heating are directly related to the shape and strength of the electromagnetic field pattern created within the tissue by the ablating antenna. As such, the shape of fields created from a MWA antenna is a consideration during the design process with reference to the clinical objectives of achieving a ablation profile to match the tumour shape. Another design consideration is the ability to efficiently transfer input power into the tissue, which can be measured by calculating the input reflection coefficient of a given probe. A high level of reflection can cause internal heating of the feed lines and within the probe structure, potentially heating tissue unintentionally or causing structural damage that affects operation of the device itself.

Several variations of MWA antenna concepts have been proposed in search of improved effectiveness, including the monopole Labonte et al. (1996), single slotted Brace (2010a), double slotted Brace (2011b), and sleeved antennas Yang et al. (2006); O’Rourke et al. (2007). Studies have investigated different probe concepts both numerically and experimentally in a comparative manner, using simulation with fixed tissue properties to calculate fundamental measurements from an isothermal contour line at the completion of ablation Ibitoye et al. (2018). Quantifying the shapes and sizes of the ablation fields extends only as far as the measurement of longitudinal length and diameter from which aspect ratio is written. However, this provides limited information for fields displaying bulges or folds. Considering that a sphere is the most desirable shape of ablation, it makes sense to use a metric quantifying how circular a two-dimensional shape is, i.e. the circularity.

The temperature dependence of the tissue dielectric properties has been shown to have a substantial influence on the simulation outcome, as changes in these properties are responsible for changing field patterns and probe performance throughout heating Ji and Brace (2011); Brace (2010b); Etoz and Brace (2018). Therefore, it makes sense to consider not only the size and shape of the electric field pattern at the beginning of ablation, but also the dynamics of how these patterns change throughout the process as the target tissue heats up.

This work presents a comparative analysis of transient changes to both shape and size of temperature and SAR fields throughout ablations simulation, examined for a variety of common probe concepts. Shape analysis metrics specific to the objective of treating a spherical tumour will be used. To achieve this, a multi-physics modelling framework on an open-source platform, wrapped within a user interface is utilized, to allow an easy investigation of the dynamic fields created from the different probe geometries and input settings. In Sect. 2 the mathematical model describing this process is established, accompanied by a description of the computational approach used and the variables measured. Following this, Sect. 2.1 presents the results of our analysis in a breakdown of each variable measured and Sect. 3 discusses the output from this work.

## Methods

Many factors influence the resulting size and shape of ablated region following MWA treatment, chief of which includes the probe geometry used and the specific tissue response to an electromagnetic field and increasing temperature.

### Coupled electromagnetic model

The electric field radiated into tissue during MWA can be computed by firstly establishing how to describe the environment from an electro-magnetics perspective. The appropriate conditions can then be applied to the Maxwell equations - a set of four equations that describe the behaviour of electric and magnetic fields and how they relate to each other. The resulting equations can be used to derive the wave equation in either the electric or magnetic field terms that are solved to yield the associated field distribution Chiang et al. (2013). What follows is a description of this process.

Firstly, the Maxwell equations can be expressed in their time-harmonic form. Here ${\textbf {E}}$ and ${\textbf {H}}$ are the electric and magnetic field intensities respectively, using *j* as the imaginary unit and $\omega$ for the angular frequency.

1 $$\begin{aligned} \nabla \cdot {\textbf {E}}&= \frac{\rho }{\varepsilon _0} \end{aligned}$$

2 $$\begin{aligned} \nabla \cdot {\textbf {H}}&= 0 \end{aligned}$$

3 $$\begin{aligned} \nabla \times {\textbf {E}}&= -j\omega {\textbf {B}} \end{aligned}$$

4 $$\begin{aligned} \nabla \times {\textbf {H}}&= \sigma {\textbf {E}} + j \omega {\textbf {D}} \end{aligned}$$

**Fig. 2:**
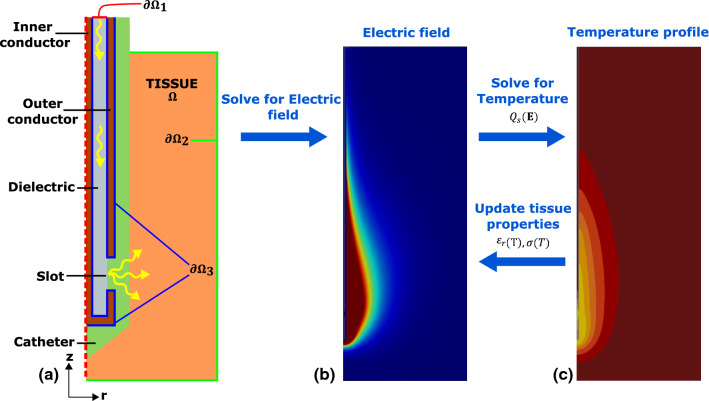
Overview of the framework used to solve the coupled problem. **a** The axisymmetric domain $\Omega$ made up of tissue and the probe constituent materials, catheter, conductors and dielectric. The three boundaries upon which conditions will be applied are shown by $\partial \Omega _1$, $\partial \Omega _2$ and $\partial \Omega _3$. EM waves propagate along the probe, from the input at $\partial \Omega _1$ through the dielectric to the slot where they are able to radiate into the catheter and tissue. **b** Typical shape of the electromagnetic field pattern created by simulated MWA probes. **c** Corresponding temperature distribution after a period of ablation

Here ${\textbf {D}}$ and ${\textbf {B}}$ are the electric and magnetic flux density respectively, with volume current density ${\textbf {J}}$. Maxwell’s equations alone cannot produce a solution. They must be supplemented by relations that describe the behaviour of a material under the influence of fields, i.e. ${\textbf {D}} = \varepsilon {\textbf {E}}$ and ${\textbf {B}} = \mu {\textbf {H}}$. Here $\mu$ is the magnetic permeability which can be expressed as the product of relative permeability $\mu _r$ and free space permeability $\mu _0$. The dielectric permittivity, $\varepsilon$, can be expressed as the product of the relative permittivity $\varepsilon _r$ and free space permittivity $\varepsilon _0$. Together these are the dielectric properties of the material.

The magnetic permeability is described as a measure of the degree to which a magnetic field can penetrate a material. In biological tissue, this can be approximated as that of the free space. The dielectric permittivity is complex-valued. The real part relates to the relative permittivity, $\varepsilon _r$, a measure of the tissue’s ability to store electrical energy whilst the imaginary part allows definition of the effective conductivity $\sigma$, representing the level of energy absorption from the electromagnetic wave. For instance, in a lossless material, the conductivity would be 0 and, hence, all energy is passed through without absorption. To express a lossy material such as tissue we assume the condition that effective conductivity is non zero: $\sigma \ne 0$. In addition, the assumption that tissue is source free can be made, meaning a value of 0 can be assigned to the charge density, $\rho = 0$. In this instance, we assume the region is both isotropic and homogeneous too.

Solving the Maxwell equations for either ${\textbf {E}}$ or ${\textbf {H}}$ can be achieved by combining them to form a vector wave equation, in this case, we formulate to solve ${\textbf {E}}$. Firstly the constitutive relations can be substituted into (3) and (4) to leave equations described by purely **H** and **E** terms. Now taking the curl of (3), the RHS has a curl of **H** term that can be substituted for (4) to leave an equation in **E** only, with wave number of free space term $k_0$.

5 $$\begin{aligned}&\nabla \times \mu _r^{-1} \left( \nabla \times {\textbf {E}} \right) -k_0^2 \left( \varepsilon _r(T) - j\frac{\sigma (T)}{\omega \varepsilon _0}\right) {\textbf {E}}=0 \end{aligned}$$

6 $$\begin{aligned}&\quad k_0 = \omega \sqrt{\mu _0 \varepsilon _0} \end{aligned}$$

Equation (5) can then be solved to compute the electric field in the tissue. As can be seen from (5), effective conductivity and relative permittivity are both temperature sensitive. Experimental studies on bovine liver samples have allowed the construction of empirical relations that estimate the changes as a function of temperature Ji and Brace (2011).

7 $$\begin{aligned} \varepsilon _r(T)&= \alpha _3 \left( 1 - \frac{1}{1 + \exp (\alpha _1(\alpha _2 - T))}\right) + 1 \end{aligned}$$

8 $$\begin{aligned} \sigma (T)&= \beta _3 \left( 1 - \frac{1}{1 + \exp (\beta _1(\beta _2 - T))}\right) \end{aligned}$$

Here, $\alpha _{1-3}$ and $\beta _{1-3}$ are the regression coefficients found in Ji and Brace (2011) and *T* is the temperature (°C). Both properties express a decreasing trend with a increasing temperature, reducing the material’s wave impedance through a proportional relation between the two. This is responsible for the changing reflection coefficients throughout ablation as seen in the results to follow.

Three types of boundary conditions can be distinguished for the electromagnetic problem, as indicated in Fig. 2a. The input boundary to the probe, $\partial \Omega _1$, must be handled in such a way that incoming microwaves can be prescribed, whilst reflected microwaves are absorbed. Waveguide port boundary conditions (WPBC) have been described as an effective way to achieve this Lou and Jin (2005). Assuming that only transverse electromagnetic modes propagate within the coaxial structure of the antenna Chaber et al. (2017) this boundary condition can be written as,

9 $$\begin{aligned} {\textbf {n}}\times \left( \nabla \times {\textbf {E}} \right) = \gamma _0\left[ {\textbf {n}}\times \left( {\textbf {n}}\times {\textbf {E}}\right) \right] - 2\gamma _0{\textbf {E}}_{inc} \qquad \text{ on } \ \partial \Omega _1, \end{aligned}$$

consisting of a surface’s normal vector, ${\textbf {n}}$, complex propagation constant $\gamma _0 = jk_0\sqrt{\varepsilon _r\mu _r}$ of the waves, and an exciting electric field incident to the surface, ${\textbf {E}}_{inc}$, that will be passed into the probe.

For calculating the magnitude and distribution of the incoming wave, Poynting’s theorem - describing the conservation of energy for an electromagnetic-field is used. For a coaxial structure with dielectric inner radius $r_i$ and outer radius $r_o$, wave impedance of dielectric within probe *Z* and input power *P*, the maximum amplitude of field strength $A_0$ can be derived as:

10 $$\begin{aligned} A_0 = \sqrt{\frac{PZ}{\pi \text{ ln }\left( \frac{r_0}{r_i}\right) }} \end{aligned}$$

with transverse electromagnetic mode field components assumed to act solely in the ${\textbf {r}}$ direction and take the form

11 $$\begin{aligned} {\textbf {E}}_{inc}(r) = {\textbf {r}}\frac{A_0}{r}e^{j k r} \end{aligned}$$

where *r* here is a radial coordinate between the bounds of $r_i$ and $r_o$. Metallic surfaces, such as those on the inner and outer conductors, are treated as perfect electrical conductors (PEC) meaning that on these surfaces the electric field exists in the tangential directions only.

12 $$\begin{aligned} {\textbf {n}}\times {\textbf {E}} = {\textbf {0}} \qquad \text{ on } \ \partial \Omega _3 \end{aligned}$$

Application of an absorbing boundary condition around the edges of the tissue converts this from an unbounded problem to one on a finite domain. Primarily this boundary should absorb any incident wave without causing undesirable reflection back into the tissue. For this purpose a similar equation to the WPBC (9) is defined, excluding excitation of the input wave

13 $$\begin{aligned} {\textbf {n}}\times \left( \nabla \times {\textbf {E}} \right) = \gamma _0\left[ {\textbf {n}}\times \left( {\textbf {n}}\times {\textbf {E}}\right) \right] \qquad \text{ on } \ \partial \Omega _2. \end{aligned}$$

The energy distribution within the tissue can be modelled using the bioheat equation, allowing transient effects to be examined. Note that the influences of blood perfusion and metabolism have been excluded,

14 $$\begin{aligned} \rho C \frac{\partial T}{\partial t} = \nabla \cdot (k_l\nabla T) + Q_s({\textbf {E}}) \qquad \text{ in } \ \Omega . \end{aligned}$$

Here, $\rho$, *C* and $k_l$ are the density, specific heat capacity and thermal conductivity of liver tissue. Coupling with the electromagnetic solutions is established through $Q_s$, the thermal source from the MWA probe. The heat generated from the action of dipole rotation in the presence of an electromagnetic field is captured by the equation

15 $$\begin{aligned} Q_{s}({\textbf {E}}) = \frac{\sigma |{\textbf {E}}|^2}{2}. \end{aligned}$$

On the external surfaces of the tissue boundary, $\partial \Omega _2$, an insulating condition is set through

16 $$\begin{aligned} (k_l\nabla T)\cdot {\textbf {n}} = 0 \qquad \text{ on } \ \partial \Omega _2. \end{aligned}$$

Therefore, the non-linear coupled system can be summarised by the equations:

17a $$\begin{aligned} \nabla \times&\beta \nabla \times {\textbf {E}} + \alpha (T) {\textbf {E}} = {\textbf {S}}&\text{ in } \ \Omega , \end{aligned}$$

17b $$\begin{aligned} {\textbf {n}}\times \left( \nabla \times {\textbf {E}} \right)&= \gamma _0\left[ {\textbf {n}}\times \left( {\textbf {n}}\times {\textbf {E}}\right) - 2{\textbf {E}}_{inc}\right]&\text{ on } \ \partial \Omega _1, \end{aligned}$$

17c $$\begin{aligned} {\textbf {n}}\times \left( \nabla \times {\textbf {E}} \right)&= \gamma _0\left[ {\textbf {n}}\times \left( {\textbf {n}}\times {\textbf {E}}\right) \right] \qquad&\text{ on } \ \partial \Omega _2 \end{aligned}$$

17d $$\begin{aligned} {\textbf {n}}\times {\textbf {E}}&= 0&\text{ on } \ \partial \Omega _3, \end{aligned}$$

17e $$\begin{aligned} \rho C \frac{\partial T}{\partial t}&= \nabla \cdot (k_l\nabla T) + Q_s({\textbf {E}})&\text{ in } \ \Omega , \end{aligned}$$

17f $$\begin{aligned} (k_l\nabla T)\cdot {\textbf {n}}&= 0 \qquad&\text{ on } \ \partial \Omega _3, \end{aligned}$$

where $\Omega$ denotes the computational domain of interest at this stage $\subset R^3$, whilst $\partial \Omega _1$, $\partial \Omega _2$ and $\partial \Omega _3$ in Fig. 2 represent the Dirichlet, Neumann and mixed boundaries respectively. Here the grouped coefficients are, $\beta = \frac{1}{\mu _r},\quad \alpha = -k_0^2\left( \varepsilon _r(T) - \frac{j\sigma (T)}{\omega \varepsilon _0}\right) , \quad {\textbf {S}}= 0$.

### Computation

The geometry of a basic single-slotted MWA probe consists mainly of a coaxial structure, an inner conductor and outer conducting layer separated by a dielectric material through which the transverse electromagnetic waves are able to propagate, see Fig. 2a. A short gap exists in the outer conductor around it’s entire circumference, through which the electromagnetic signal is able to propagate out of the dielectric and into the surroundings. All of this structure is then stored within a catheter which allows for safe insertion. Probe geometries considered in this analysis are rotationally symmetric, allowing a reduced domain through exploitation of this axisymmetry.

As described in (), this multi-physics problem requires coupling of an electromagnetic wave and bioheat equation, both of which contain variables that have a degree of temperature sensitivity that are updated as the simulation steps through time. This coupling is summarised in Fig. 2b and c. The open source NGSolve library was chosen as a foundation to solve this coupled system by FEM hp finite elements Schöberl (2014). Geometries of each probe and surrounding tissue domain are created and meshed in the GMSH software package. An unstructured mesh of triangular elements is created with refinements near the slot in the antenna where higher gradients within the electric field are observed. The electromagnetic equation is discretised by third order H(Curl) elements and second order Lagrange elements were used for discretising the bioheat equation on the same mesh using NGSolve Geuzaine and Remacle (2009). A weak coupling exists whereby material properties sensitive to temperature are updated at the end of each time step before resolving the electromagnetic problem again, this framework is summarised in Fig. 2b and c.

**Fig. 3:**
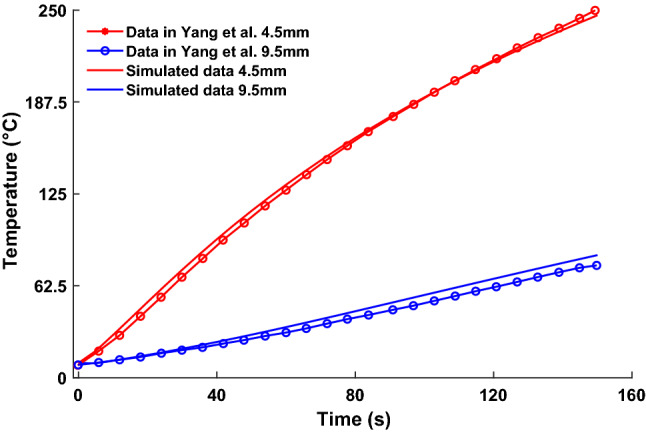
The comparison of simulated tissue temperature using the coupled model presented, against an established numerical solution found in Yang et al. (2007). A common power setting, probe and domain size is used by both simulations for this validation

As a validation of the accuracy of the coupled model, existing work published by Yang et al. (2007) is used for comparison, similarly to other publications Keangin et al. (2011). As such, a single slotted probe geometry was created and parameters set to match those used in the comparative study, then an ablation of 150 s at 50W power is simulated at 5 s time steps. Temperature values within the tissue are taken at specific radial distances from the probe, at depth level with the center of the slot. Figure 3 shows these temperature changes within the tissue throughout ablation and displays good agreement between the model presented and that of the established model in literature.

### Design variables

#### Reflection coefficient

If $*$ indicates the complex conjugate of a variable, the reflection coefficient of a probe is given by

18 $$\begin{aligned} \mathrm {S_{11}} = \Gamma = \frac{\int _{\partial \Omega _1}\left( {\textbf {E}}-{\textbf {E}}_{inc}\right) \cdot {\textbf {E}}^*_{inc} \ d(\partial \Omega _1)}{\int _{\partial \Omega _1}{} {\textbf {E}}_{inc}\cdot {\textbf {E}}^*_{inc} \ d(\partial \Omega _1)}, \end{aligned}$$

which is a measure of how well-matched the impedance of the tissue is to the characteristic impedance of the probe itself. A closely matched impedance will translate to a larger proportion of the energy supplied to the probe being transferred into the tissue, and therefore more energy absorption. In addition to negating issues associated with high power reflection.

A coaxial MWA probe can be considered as a one port system, where the S11-parameter is equal to the reflection coefficient $\Gamma$. Calculation is possible in terms of the total electric field, ${\textbf {E}}|_{\partial \Omega _1}$, and exciting electric field input, ${\textbf {E}}_{inc}|_{\partial \Omega _1}$, measured on the antenna input surface $\partial \Omega _1$ Gas (2018).

#### Shape analysis

Assuming the ablation shape, in this analysis, is desired to be as spherical as possible in order to treat a spherical tumour. Temperature and absorption field shapes are formed from the simulated data and their shapes are subsequently analysed, calculating a shape metric in 2D that is equivalent to measuring sphericity in 3D.

**Fig. 4:**
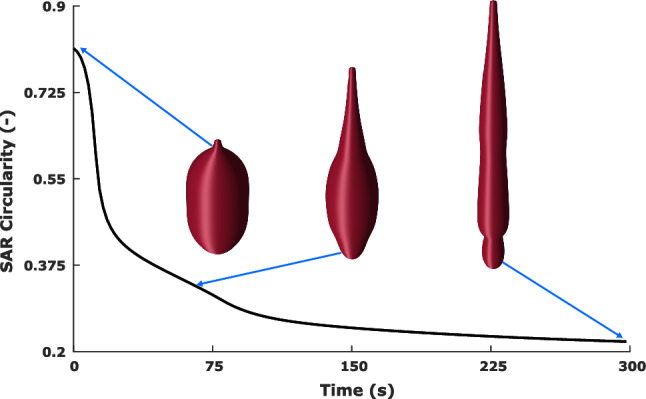
Absorption patterns segmented by the 1kW/m$^{3}$ isocontour throughout the ablation simulation, annotated against the absorption circularity curve

When analysing the shape and size of these distributions, it is helpful to define an enclosed boundary. This boundary is identified by setting a isocontour value for each specific field variable. Once these boundaries are defined in the axisymmetric data, the shape can be segmented for analysis. Figure 4 demonstrates how the SAR field pattern deforms from its original shape when heated.

Two dimensional area of each shape can be calculated for exact measure of size. A descriptor of shape circularity using measures of area and perimeter

19 $$\begin{aligned} \mathrm {Circularity} = \frac{4\pi \ \mathrm {Area}}{\mathrm {Perimeter}^{2}}, \end{aligned}$$

this yields a result that is constant regardless of the scale of shape, and with values vary relative to 1 which represents a perfect circle. Figure 4 demonstrates how this measurement might change.

#### Probe concepts

**Fig. 5:**
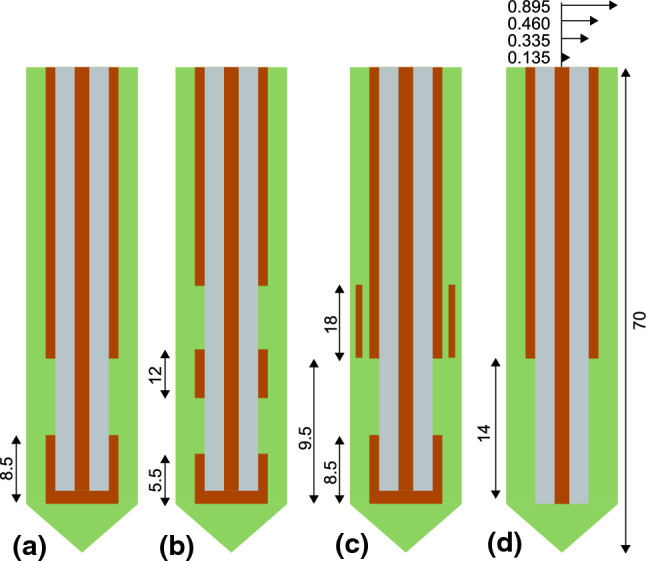
Illustrations of geometry for each of the probe concepts with specific geometry (mm). **a** single slot (SS), **b** Dual slot (DS), **c** Sleeve Single slot (SSS) and **d** Monopole (M)

Several variations for the design of MWA probes have been proposed and studied. Four main concepts could be distinguished that will be simulated and compared in this work. These include the monopole (M) probe structure Fig. 5a, which maintains the main coaxial body but with a section of the outer conductor removed towards the end. The single slotted (SS) probe Fig. 5b described prior in the methods section, a dual slotted (DS) probe Fig. 5c with two gaps in the outer conductor separated by a fixed length and a sleeve single slotted (SSS) probe Fig. 5d, containing the same coaxial geometry of the single slot but with an additional layer of conducting material within the catheter.

The DS probe effectively has two sources that radiate out from the antenna, one from each slot, these two waves interact destructively around the upper region of the upper slot to reduce elongation of the electric field pattern along the outside of the outer conductor. Reducing this elongation aims to create more concentrated absorption patterns that are closer to the spherical ideal. A SSS probe aims to achieve the same outcome by introducing a sheath of conducting material within the catheter that surrounds the coaxial structure, but maintaining separation from the outer coaxial layer itself. These designs have been shown to confine the absorption pattern towards the probe tip.

For consistency, common dimensions are used for the coaxial and catheter parts of all probes simulated. The outer radii of coaxial components, in mm, are as follows: inner conductor (0.135), dielectric (0.335), outer conductor (0.46) and catheter (0.895). All slotted probes use a slot width of 1 mm and the sleeve component has a thickness of 0.15 mm. Probe specific dimensions are described in Fig.  5. The length of each probe is simulated as 70 mm in a tissue domain of 80 mm high and a radius of 40 mm.

## Results

Simulations use a microwave frequency of 2.45 GHz and with an input power of 30W, whilst each ablation simulates 300 s. The reflection coefficient, SAR and temperature distribution are all calculated throughout and subsequently used for further shape analysis. Repeat simulations are made using constant tissue properties and plotted alongside to emphasise the difference this can make.

**Fig. 6:**
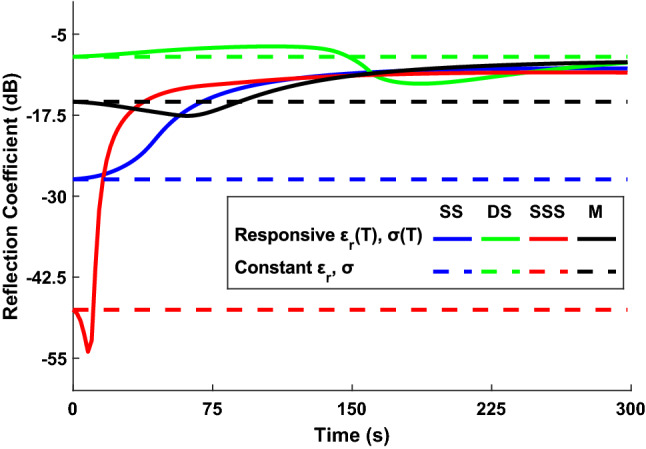
Graphic highlighting the significant changes in the reflection coefficient throughout ablation across the variety of probe designs, demonstrating the need to capture the dynamic changes in coefficients as opposed to treating them as constant

**Fig. 7:**
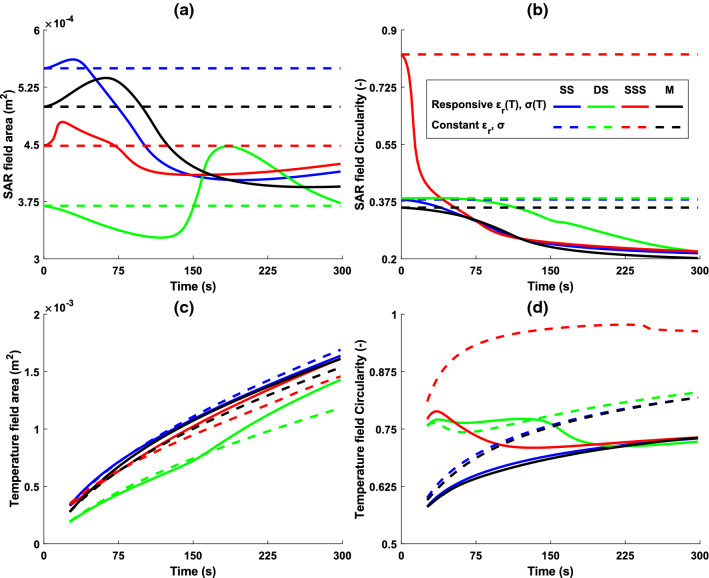
Results presented for SAR **a** area and **b** circularity, then Temperature **c** area and **d** circularity. The influence of temperature sensitive dielectric tissue properties is apparent when compared with simulated results using constant dielectric values

Figure 6 shows the temporal changes in reflection coefficient of each probe design through ablation. Initially, with the dielectric properties of liver tissue at 37°C, the calculated reflection coefficients from least to most efficient are: DS -8.5dB, M -15dB, SS -27dB closely followed by SSS -47dB.

M, DS and SSS probes all display reflection coefficient changes and a minimum value of reflection at some point, showing that as the surrounding tissue undergoes changes to its dielectric properties brought on by temperature increase, the impedance match to each of these probes improves up to a point of best coupling (minimum reflection) before becoming less well matched again. The timing, minimum value and gradient either side of the trough are different for each probe. The most extreme reduction is visible for the SSS probe, displaying a sharp reduction in the first 10 s, upon which the minimum value -54dB is measured followed immediately by a steep increase that remains until around 30 s before plateauing, finishing at -11dB.

Changes in the reflection coefficient of the DS probe remain fairly constant apart from a dip at around 150 s, shortly after a minimum value -13dB is observed, followed by a shallow increase that lasts for the remainder of the simulation, ending with reflection coefficient of -10dB. This particular M probe shows a subtle drop in reflection coefficient from its starting value, with a minimum recorded at 62 s of -18 dB before increasing gradually to a final value of -10 dB. As the temperature increases in the tissue, dielectric properties decrease and consequently the impedance decreases. This changeable impedance over time is responsible for the dynamic variations in the reflection coefficients during ablation. Since the largest changes generally occur in the earlier stages of ablation, this suggests that tissue closest to the probe (where SAR values are high and temperature increase occurs rapidly) mostly determines the efficiency of energy transfer. Towards the end of ablation the tissues closer to the probe are undergoing less changes to their properties.

The size of the threshold SAR field created for each probe design is captured in Fig. 7.a, which displays the data for total area above an SAR threshold of 1kW/m$^{3}$. At the first instance of power being supplied to the probes, the segmented areas in increasing order are 3.70 $\times 10^{-4}$m$^{2}$ for DS, 4.47 $\times 10^{-4}$m$^{2}$ SSS, 4.99 $\times 10^{-4}$m$^{2}$ for M and 5.49$\times 10^{-4}$m$^{2}$ for SS. Whilst the presented results in isolation already contain useful information, it is the collective that provides a more complete picture of the diverse transient changes observed between probes. A common theme shared by all probes is an increase in SAR area measured at or shortly proceeding the reduced reflection coefficient seen in Fig. 6, and reduced area seen when the reflection coefficient increases. This is most clearly illustrated for the DS probe with an obvious increase around 150 s. The shape analysis of the SAR pattern is presented using the circularity metric, as shown in Fig. 7.b. Initially, the SSS design has the highest circularity of 0.81, with the three other concepts closely matched in the range 0.35–0.38. All probes display a trend of reducing circularity throughout ablation, although with different gradients, the higher circularity from SSS probe reduces at a greater rate. Final measures across all probes spans a small range between 0.20 and 0.22. The same circularity analysis is performed on the temperature field. Unlike their SAR counterparts the 50°C thresholded thermal areas show a simple increase throughout the ablation process, which is observed consistently for all probes as illustrated in Fig. 7c. Initial calculated values show the DS probe has the smallest area early on with 1.9$\times 10^{-4}$m$^{2}$, followed by M at 2.8$\times 10^{-4}$m$^{2}$ with SS and SSS around 3.3$\times 10^{-4}$m$^{2}$. The M, SS and SSS follow similar values throughout and end closely in the range 16.1–16.4$\times 10^{-4}$m$^{2}$, with the DS probe following the same trend but at a lower value throughout, finishing at 14.3$\times 10^{-4}$m$^{2}$. The temperature shape analysis is shown in Fig. 7d. The SS and M probes show curves of an asymptotic nature with their starting values of 0.58 and end values of 0.73. The SSS and DS probes show different characteristics with increasing circularity values for a period before reaching a maximum and reducing for the remainder of the ablation. Potentially as a result of the markedly different circularity of SAR patterns when compared to the SS and M probes, as evident in Fig. 7c.

## Discussion

This study has highlighted the effect of the dynamically changing dielectric and thermal properties on the shape and size of the electromagnetic and thermal fields during ablation. The work shows a direct comparison of 4 typical probe designs and builds on a recent MWA probe comparison study Ibitoye et al. (2018) which assumed constant material properties. When comparing dynamically changing tissue properties alongside the corresponding results with fixed properties, the importance of including such temperature dependencies within simulations is clear to see.

The reflection coefficients for all antenna designs showed a range of efficiencies throughout ablation. Hence, although it was found that the addition of a sleeve to the SS design (denoted as SSS) reduces wave reflections at the start of ablation consistent with literature Brace (2010a); Ibitoye et al. (2018), the transient responses of the tissue significantly change this behaviour. Three of the designs display an increasing reflection coefficient compared to their starting value. This reinforces the need to take account of the tissue property changes during probe design cycle to accurately predict undesirable heating along the probe caused by reflected power. Results here suggest the magnitude of these changing reflections is markedly different between the designs, and although the SSS at times has the lowest levels of reflection, it is also the most variable throughout ablation, suggesting a higher sensitivity to changes in the tissue dielectric properties incurred as heating progresses.

The importance of transient tissue reponses is further highlighted in the temperature analysis where the shape (circularity) of the ablation areas vary greatly, whilst the overall heated areas grow similarly for each probe. For example, when the material properties are taken constant, the SSS design appears to create much more circular absorption patterns outperforming the other probes as it more closely matches the typically spherical shape of tumours. However, this effect disappears the moment variable tissue properties are introduced, which make all probes perform equally (in terms of shape) at the end of ablation. These results could question the use of a sleeve in a probe design, although further analysis and probe optimisation is required to provide a conclusive answer. The overall trend observed between the probes is that there are some profound performance differences between them in the early stages of ablation that seem to disappear towards the end of ablation.

Even though results as presented are useful a few limitations can still be identified. The transient behaviour in these simulations is strongly dependent on the coupling between the electromagnetic and thermal problems, i.e. the material models used. More detailed and robust material models are required to get quantitative predictive ablation models. This includes the physiological changes that occur during tissue ablation, such as perfusion (in vivo) or tissue shrinkage when exposed to temperature increase.

Also the geometries used in these simulations are chosen based on literature, but without consideration of manufacturability. This was done so that the probe diameter was consistent across all probes tested and for a more intuitive comparison. Optimisation approaches such as presented in Etoz and Brace (2018) are recommended to explore the potential of different probe types.

As this study has demonstrated, a thorough study of the dynamics is key to understanding tissue ablation. This might be emphasised even further if one would consider the effects of transient thermal loading on cellular damage models.
